# Improving Adherence to a Mediterranean Ketogenic Nutrition Program for High-Risk Older Adults: A Pilot Randomized Trial

**DOI:** 10.3390/nu15102329

**Published:** 2023-05-16

**Authors:** Julia L. Sheffler, Dimitris N. Kiosses, Zhe He, Bahram H. Arjmandi, Neda S. Akhavan, Kamelia Klejc, Sylvie Naar

**Affiliations:** 1 Center for Translational Behavioral Science, College of Medicine, Florida State University, Tallahassee, FL 32306, USA; kk22n@med.fsu.edu (K.K.);; 2 Weill Cornell Institute of Geriatric Psychiatry, Weill Cornell Medical College, White Plains, NY 10605, USA; 3 School of Information, Florida State University, Tallahassee, FL 32306, USA; zhe@fsu.edu; 4 College of Health and Human Sciences, Florida State University, Tallahassee, FL 32306, USA; barjmandi@fsu.edu (B.H.A.); nakhavan@fsu.edu (N.S.A.)

**Keywords:** Mediterranean, ketogenic, nutrition, diet

## Abstract

(1) Background: Mediterranean ketogenic nutrition (MKN) may directly target multiple neurobiological mechanisms associated with dementia risk in older adults. Despite its promise, this type of nutrition can be challenging to learn and adhere to in a healthy manner. Our team used the National Institutes of Health Obesity Related Behavioral Intervention Trials (NIH ORBIT) model to develop and pilot a program to help older adults with memory concerns use MKN. (2) Methods: Using a two-arm, randomized design, we evaluated an MKN Adherence (MKNA) program compared to an MKN education (MKNE) program (*N* = 58). The primary difference between study arms involved the use of motivational interviewing (MI) strategies and behavior change techniques (BCT) only in the MKNA arm. Participants were included if they evidenced subjective memory concerns or objective memory impairment on the Montreal Cognitive Assessment (Score 19 ≤ 26). Primary outcomes examined included feasibility, acceptability, adherence, and clinical outcomes associated with the program. (3) Results: Overall, there was relatively high program completion in both groups, with 79% of participants completing the 6-week program. The recruitment protocol required adjustment but was successful in reaching the target sample size. Retention (82%) and session attendance (91%) were higher in the MKNA arm compared to the MKNE (retention = 72%; attendance = 77%). Overall, most participants in both groups rated the program as “excellent” using the client satisfaction questionnaire. Participants in the MKNA arm evidenced higher objective and self-reported adherence to MKN during the 6-week program. Further, there was some evidence of clinical benefits of the program, although these effects diminished as adherence decreased in the 3 months follow-up. (4) Discussion: This pilot trial demonstrated that the MKN program incorporating MI and BCT strategies may better engage and retain participants than a nutrition education program alone, although participants in both groups reported high satisfaction.

## 1. Introduction

Research shows that modifiable factors account for up to 40% of Alzheimer’s disease and related dementia (ADRD) risk [1], demonstrating a critical need to develop scalable interventions to address ADRD risk. In particular, behavioral interventions targeting lifestyle in mid- and late-life may address multiple modifiable risks [2,3]. For ADRD risk, Mediterranean ketogenic nutrition (MKN) holds substantial promise as a non-pharmacologic therapeutic strategy for addressing multiple modifiable risks associated with ADRD [4]. The Mediterranean and ketogenic diets separately have demonstrated benefits for brain health that may reduce risk for cognitive decline [5,6]. Specifically, a recent systematic review of the literature examining the relationship between the Mediterranean diet and brain health found that there is strong evidence that adherence to a Mediterranean diet is protective against cognitive decline and associated with improved global cognition in middle-aged and older individuals [5]. While fewer clinical trials have examined the effects of a ketogenic diet, there is growing evidence that it provides significant benefits for cognition in individuals with mild cognitive impairment (MCI) and mild to moderate ADRD [7]. Recent research has found that a modified Mediterranean ketogenic diet may provide additional benefits for multiple biomarkers associated with ADRD in addition to cognitive functioning [8,9].

Multiple mechanisms between these diets and ADRD have been proposed. For the Mediterranean diet, there is evidence that benefits may be derived through increased fruit and vegetable intake in addition to high intake of monounsaturated fats, which may reduce inflammation and oxidative stress, in addition to cardiovascular risks for ADRD [4]. Mechanisms linking a ketogenic diet with ADRD are primarily related to the role and function of ketone bodies, which are created when an individual consumes a very low carbohydrate and high fat diet that leads to a metabolic shift from using glucose to ketones for energy. Research shows that ketones provide a more efficient cerebral energy source, improve mitochondrial function, and reduce oxidative stress and inflammatory processes [4]. Further, ketogenic diets have shown promise in enhancing insulin sensitivity and regulation, which is increasingly recognized as a significant risk for ADRD development [10]. Although it is important to note that there are some mixed findings on ketogenic diets in relation to heart health in animal models [11,12], the majority of evidence supports the safety and efficacy of consuming high quality MKN for brain and cardiometabolic health [7,13,14]. Given the existing evidence and early clinical trials, there is reason to conclude that a low-carbohydrate diet, high in monounsaturated fats, vegetables, fruits, and whole foods, may address multiple pathological mechanisms associated with ADRD risk.

Despite promising evidence of the effectiveness of these diets, there are multiple societal and individual-level factors affecting the integration of this information into daily practice. First, nutrition recommendations are frequently changing, and specialized ways of eating are often viewed as “fad diets.” Recently, these problems have been exacerbated by highly processed, expensive products labeling themselves as “keto” or “heart healthy,” without evidence to support claimed health benefits. Thus, misinformation about the health benefits of different foods is highly prevalent, and many individuals do not have the information needed to make choices that will promote brain health. Another challenge, common to many lifestyle interventions and diets, is the issue of long-term adherence. Mediterranean and ketogenic diets may be particularly challenging due to the greater divergence from a traditional Western diet, as well as perceived costs of these “healthier” foods. Further, adherence is influenced by a range of personal characteristics, such as enjoyment from eating unhealthy foods, feeling deprived, and eating due to stress or anxiety [15].

The success of MKN in addressing ADRD risk rests on the ability to develop behavioral interventions that provide individuals with the education, support, and skills needed for making this lifestyle change in a way that can be easily disseminated to the larger population. The National Institute of Health-funded Obesity Related Behavioral Intervention Trials (ORBIT) model provides a framework for developing and testing behavioral interventions for chronic diseases [16]. Further, ORBIT provides a pathway for refining and tailoring interventions to address community needs and improve scaling and dissemination of effective interventions. Using the ORBIT model, our team established the proof-of-concept for a community-focused MKN program that uses motivational interviewing strategies and behavior change techniques (MI-BCT) to enhance adherence [17].

To address these challenges, our team developed a nutrition program designed for older adults with cognitive concerns and possible MCI. This 6-week group program provides participants with in-depth information about using MKN, framing this shift as a change in nutrition versus short-term dieting. Participants are provided with basic nutrition education for broadly understanding and evaluating the food they eat and purchase, while providing a supportive environment and additional skills training for overcoming personal challenges. Specifically, we incorporated principles of MI and BCTs to help participants identify their own values and reasons for making a lifestyle change, while providing structured skills practice for overcoming long-term obstacles. BCTs are often considered the ‘active ingredient’ in behavioral interventions that influence mechanisms of action [18]. Our team used the NIH ORBIT framework for developing behavioral interventions to address chronic conditions [16] and has previously established the proof-of-concept for this MKN adherence (MKNA) program [17].

Using the ORBIT framework, the current study was a feasibility pilot (Phase 2b) designed to evaluate the MKNA program in a larger sample with a goal of assessing whether the MI-BCT components are effective in improving adherence or require additional refinement. Further, the study aimed to confirm the feasibility and acceptability of the program, as well as evaluate important clinical outcomes, such as cognition and cardiometabolic markers. A two-arm, pilot clinical trial was completed with a total of 58 participants who were randomized to an MKNA arm or an MKN education (MKNE)-only arm.

## 2. Materials and Methods

### 2.1. Participants and Design

**Figure 1:**
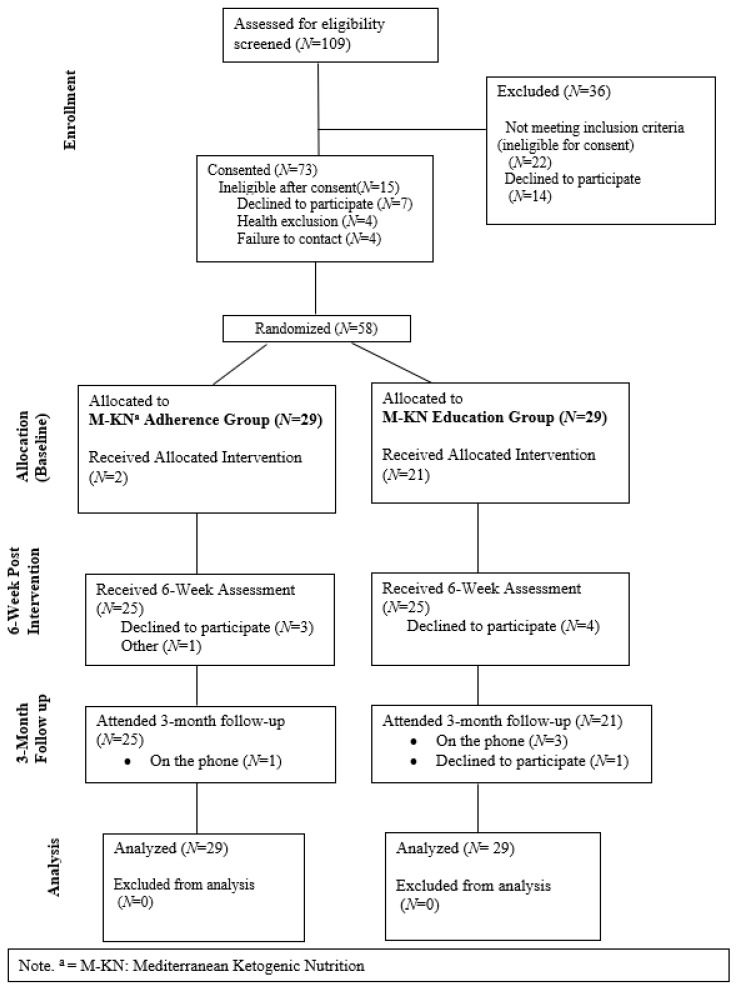
CONSORT Diagram.

Individuals aged 60–85 with possible mild cognitive impairment were recruited from a senior health clinic, the local community, and from a participant registry for participation in this two-arm randomized pilot trial. A telephone screen was completed with 109 individuals to evaluate initial eligibility. Participants were scheduled to complete the informed consent process if they were between the ages of 60–85, interested in the described nutrition program, had internet access, were English-speaking, and had a score of >1 on the Memory Complaint Scale (MCS) or a Telephone Montreal Cognitive Assessment (TMoCA) adjusted score between 19 and 26. Participants were excluded if they reported any of the following: (1) major or unstable health conditions, including Type 1 diabetes, (2) major psychiatric conditions (e.g., schizophrenia), (3) being under-weight based on a BMI < 19 kg/m^2^, (4) taking medications that may be contraindicated (e.g., insulin, MAOIs, immunosuppressants), or (5) being deemed ineligible based on cognitive status (adjusted TMoCA < 19). Based on these criteria, 73 individuals were scheduled to be consented; see Figure 1 for the full CONSORT diagram. A final eligibility check was completed at the baseline appointment after reviewing health conditions and medications with the participant. Participants were randomized at the end of the baseline appointment. All procedures were reviewed and approved by the Florida State University Institutional Review Board. In total, 58 participants were randomized to either the MKNA arm (*N* = 29) or an MKNE arm (*N* = 29).

All participants completed in-person assessments at baseline (within 2-weeks of beginning the program), at 6-weeks (the final week of the program), and at 3-months post-intervention. Additionally, participants used at-home urinalysis test strips to assess ketone levels daily, logged at least three days of food and drink intake, and completed weekly surveys to input this and other relevant health and psychosocial information during the 6-week program. Blood draws were completed at baseline and 6-weeks, and brief neuropsychological testing was completed at each in-person appointment in addition to standardized health and psychosocial measures of functioning. Additionally, at the 6-week and 3-month post-intervention assessments, participants completed exit interviews about their experience in the program and study in order to identify barriers and facilitators for refining the program and study protocol to enhance participants’ experience.

#### 2.1.1. MKNA Arm

The MKNA program involves seven 1 h virtual group meetings via a HIPAA-compliant Zoom meeting across six weeks. Each meeting was led by a clinical psychologist and nurse practitioner with expertise in functional medicine and involved nutritional education and psychoeducation. Each session dedicated approximately half of the time toward diet and nutrition content and the second half of the session toward MI-BCT components. All sessions were led based on a structured PowerPoint presentation to increase fidelity. In regard to the nutrition components, the first two sessions introduced MKN, how to track macronutrients, and how to use the program resources (e.g., recipes, informational handouts, and individualized calculators). In weeks two through six, participants were asked to attend weekly sessions to learn about topics related to MKN, eating healthy on a budget, and trouble-shooting questions about the diet. Participants were provided weekly with individualized gram recommendations for fats, proteins, and carbohydrates based on their age, sex, BMI, and activity level. Each week, these individualized recommendations titrated toward a ketogenic ratio: all participants began at a 50% fat, 25% protein, and 25% net carbohydrate recommendation and titrated up to a 70% fat, 25% protein, and 5% carbohydrate ratio. Once in a state of ketosis, however, participants were recommended to stay at the least restrictive ratio possible. Given this titration, participants were not expected to evidence urine ketones until weeks 3 or 4 of the program. Participants were provided with guidance for completing their own meal planning and preparation, allowing substantial individual differences; however, emphasis was placed on consuming the types of foods consistent with a Mediterranean diet (e.g., fish, olive oil, and unprocessed foods), while applying a ketogenic macronutrient ratio. Participants were provided with over 130 optional breakfast, lunch, dinner, and snack recipes to assist with meal planning, in addition to informational handouts, phone application recommendations, and tools for tracking macronutrients.

The second half of each session for the MKNA arm was dedicated to motivational discussions (e.g., goal setting and values) and teaching MI-BCT skills (e.g., overcoming obstacles, managing slips, and positive reappraisal). Early sessions placed greater emphasis on building motivation and then shifted toward learning basic cognitive restructuring and behavioral skills, and final sessions focused on overcoming future obstacles and maintaining motivation. Participants received a workbook with worksheets focused on learning MI-BCT strategies (e.g., problem solving and cognitive restructuring) and informational handouts. In session, examples of each skill were reviewed, and participant discussion and feedback was encouraged. Participants were asked to complete worksheets between sessions and bring these the following week to share with the group, although sharing was optional.

#### 2.1.2. MKNE Arm (Active Comparator)

Participants in the MKNE arm received the same structured nutrition education, monitoring, workbook, and MKN guidance. Sessions were similarly led by a nurse practitioner and psychologist; however, these sessions excluded the MI-BCT components in-session or in the workbook.

### 2.2. Measures

#### 2.2.1. Feasibility (Primary)

The feasibility of recruiting and retaining participants for the trial was evaluated based on participant recruitment rates, retention in the study and intervention, and session attendance. Criteria indicating success of feasibility were set at 80% for retention in the intervention and trial and at 90% for session attendance. Feasibility of our recruitment protocol and criteria was based on ability to meet our proposed study N of 60 and participant racial/ethnic representation (i.e., 50% White, 25% Black or African American, and 25% other).

#### 2.2.2. Acceptability (Primary)

The acceptability of the intervention was evaluated using items from the eight-item version of the Client Satisfaction Questionnaire (CSQ-8) [19]. Specifically, participants were asked to rate the quality of service they received (1 = Poor and 4 = Excellent) and overall how satisfied they were with the service they received (1 = Quite dissatisfied and 4 = Very satisfied).

#### 2.2.3. Adherence (Primary)

Participant adherence to the nutrition program was evaluated using participant self-rated adherence (0 = Not at all, 5 = Half of the time, and 10 = All the time, very consistent) and ketone urinalysis test strips measuring acetoacetic acid. Self-rated adherence was collected at each of the weekly online surveys and at the 3-month follow-up assessment. To evaluate self-rated adherence during the program, weekly scores were summed for analyses, with higher scores corresponding with higher adherence to MKN. Urine ketones were measured at each in-person assessment and daily at-home during the 6-week intervention. Participants were instructed on appropriate storage and use of the ketone urine test strips and provided with tracking sheets to enter their daily values. Participants were instructed to measure ketones in the morning prior to eating or drinking each day.

#### 2.2.4. Clinical Outcomes (Secondary)

Cognition. Cognitive outcomes were evaluated in-person at baseline, 6-weeks, and 3-months using the updated Repeatable Battery for the Assessment of Neuropsychological Status (RBANS) forms A, B, and C at each time point, respectively. Given the focus on evaluating cognitive domains associated with ADRD risk, the RBANS Total Score and Delayed Memory Index were examined for change across time and group differences. These standardized scores are normed based on participant age, with a score of 100 indicating average performance compared to peers. Of note, due to an administration error, one participant was missing baseline RBANS A data for the total score and Delayed Memory Index.

Cardiometabolic. Participants were instructed to fast overnight (8–10 h) prior to reporting to the laboratory in the morning for study visits. Fasted blood samples were collected at baseline and 6-weeks, while height, weight, and blood pressure were assessed at all three study visits. All venous blood samples were obtained in vacutainers with the appropriate anticoagulants. Serum and plasma were separated by centrifugation at 3500× *g* for 15 min, then aliquoted, and stored at −80 °C until analysis. Plasma samples were analyzed using a Piccolo Xpress chemistry analyzer to evaluate blood glucose and total cholesterol. Body mass index (BMI) was calculated at each time point using the Centers for Disease Control and Prevention online BMI calculator. Blood pressure was collected using a HealthSmart blood pressure monitor for the upper arm following approximately 10 min of supine rest. A1CNow+ Professional point of care test kits for hemoglobin A1c (HbA1c) were used at baseline and 6-weeks to evaluate HbA1c levels.

### 2.3. Analyses

We used intent-to-treat methods for analyses, including all individuals who were randomized with available data. It is important to note that phase II of the ORBIT model is focused on determining whether a protocol can be administered with fidelity and evaluates whether it can produce a clinically meaningful change on the behavioral and clinical risk factors. Thus, clinical rather than statistical significance is of greatest interest at this phase. Feasibility outcomes were examined using recruitment and retention rates across the full sample and separately based on intervention arm. Acceptability was evaluated based on participant ratings of the program overall, as well as independent samples *t*-tests to evaluate group differences. Given the importance of understanding adherence in this trial, this outcome was examined using multiple methods. First, we used *t*-tests to evaluate group differences in adherence based on self-report, average ketone levels across the 6-week program, and ketone levels at the 6-week in-person assessment. Additionally, we examined correlations between participant characteristics at baseline and adherence to identify variables that may be important for future program refinements and tailoring. Finally, for secondary outcomes, we used paired samples *t*-tests to evaluate change from baseline to follow-up and baseline to 3-months across groups. Further, we calculated change scores for these two time frames and used *t*-tests to examine group differences in change on secondary outcomes.

## 3. Results

### 3.1. Baseline Sample Characteristics

See Table 1 for baseline characteristics of the sample. Briefly, the mean age of the sample was 72.91 (*SD* = 6.35), and 81% of the sample identified as female. At screening, the median score on the MoCA was 25.91 and 4.24 on the Memory complaint scale, indicating possible mild cognitive impairment; however, participants scored in the average range on the RBANS A total score (*M* = 102.05, *SD* = 14.94) and on the delayed memory index (*M* = 101.60, *SD* = 16.62) at baseline.

### 3.2. Feasibility

In regard to feasibility of recruitment, of the 109 individuals who completed screening, approximately 67% met initial eligibility criteria and completed the informed consent. An additional 20% of participants were identified as ineligible based on health problems or declined to participate following the consent appointment. In total, 53% of screened participants were included in the trial. Of these, 79% identified as white; 16% identified as black or African American, and 5% identified as “other.” Thus, while we met our feasibility success criteria for total sample size and recruitment rates, poor participant representation will need to be addressed in future trials. In regard to retention rates, across both arms 86.2% of the sample remained in the study until the 3-month endpoint, and 78% of the sample completed the intervention program. Higher rates of retention were evident in the MKNA arm, where 83% of the sample completed the program and 89.6% of the sample completed the study, compared to 72% and 82.7%, respectively, in the MKNE arm. Further, participants in the MKNA arm attended the program sessions more consistently, with 90.7% attendance in the MKNA arm compared to 76.7% attendance in the MKNE arm. Thus, only the MKNA arm achieved feasibility success criteria for retention.

### 3.3. Acceptability

Across both arms, 65.1% of participants rated the service as “excellent,”; 32.6% rated the service as “good,” and 2.3% rated the service as “fair,” and 60% of the sample reported that they were “very satisfied” with the service. The MKNA arm tended to rate the quality of the service (*M* = 3.65, *SD* = 0.57) and their satisfaction (*M* = 3.63, *SD* = 0.49) higher than the MKNE group for quality (*M* = 3.6, *SD* = 0.50) and satisfaction (*M* = 3.38, *SD* = 0.87); however, these differences were not statistically significant. Thus, while participants appeared to prefer the MKNA program, both the MKNA and MKNE programs were highly acceptable to participants.

### 3.4. Adherence

Based on weekly self-reported adherence across the 6-week program, on average participants reported adhering half of the days to MKN (*M* = 5.24, *SD* = 2.47). Across both arms, 47% reported adhering more than half the days (>5/10). Participants in the MKNA arm (*M* = 5.97, *SD* = 2.53) reported significantly higher adherence across the 6-weeks than participants in the MKNE arm (*M* = 4.49, *SD* = 2.21; *t*(47) = 2.18, *p* = 0.034). Similarly, participants in the MKNA group *(M* = 17.32, *SD* = 14.41) reported more days with greater than trace level of urine ketones compared to the MKNE group (*M* = 10.54, *SD* = 12.83), although this difference was not statistically significant (*t*(47) = 1.74, *p* = 0.089). See Table 2 for a summary of group differences in adherence.

By the 3-month post-intervention point, only six participants evidenced measurable levels of urine ketones during the in-person assessment; however, 42% of the total sample continued to report adhering to MKN at least half of the days since ending the program. At the 3-month assessment, there were no significant differences in self-reported adherence between the MKNA (*M* = 3.50, *SD* = 2.99) and the MKNE arms (*M* = 3.35, *SD* = 2.77; *t*(47) = 0.184, *p* = 0.854).

Including screening and baseline participant characteristics (i.e., age, sex, race, education level, MoCA total score, RBANS A total scale score, and MCS), we examined bivariate correlations with measures of adherence at 6-weeks and 3-months. Of these characteristics, only age and MoCA total score were significantly associated with measures of adherence. Specifically, younger participants were more likely to report greater adherence to MKN across the 6-week program (*r* = −0.346, *p* = 0.015), and participants with higher MoCA scores at baseline were more likely to reported more days with greater than trace levels of ketones during the program (*r* = 0.293, *p* = 0.041).

### 3.5. Clinical Outcomes

For cognition, across both arms there was statistically significant improvement from baseline to 6-weeks for the RBANS total scale score (*t*(47) = −2.04, *p* = 0.047) and the Delayed Memory Index score (*t*(47) = −2.09, *p* = 0.042). There were no significant changes in HbA1c, blood glucose, or total cholesterol levels from baseline to 6-weeks. Systolic blood pressure significantly decreased from baseline (*M* = 136.66, *SD* = 20.72) to 6-weeks (*M* = 130.22, *SD* = 20.81; *t*(49) = 3.02, *p* = 0.004). BMI also significantly decreased from baseline (*M* = 27.57, *SD* = 5.70) to 6-weeks (*M* = 27.02, *SD* = 5.42; *t*(49) = 2.12, *p* = 0.039). When examining change scores for between group difference from baseline to 6-weeks, there were no statistically significant differences between groups on any of the clinical outcomes (*p* > 0.05).

**Figure 2:**
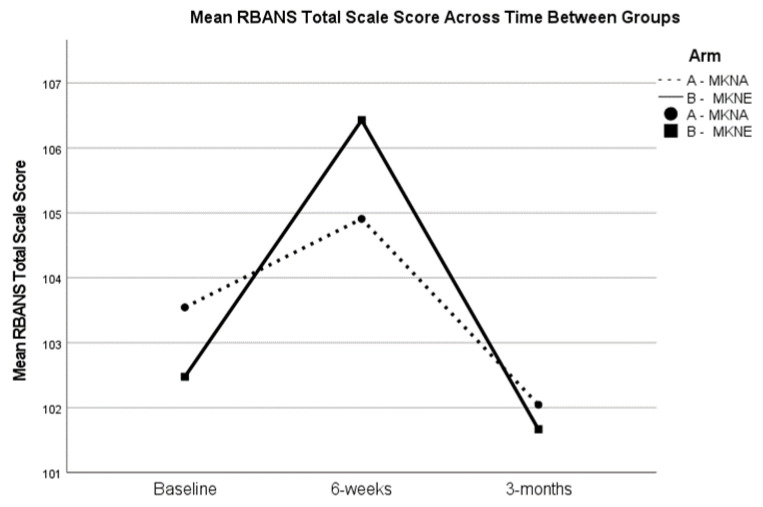
Changes in RBANS Total Scores. RBANS = Repeatable Battery for the Assessment of Neuropsychological Status Indexes; Changes in mean RBANS total scale score for the MKNA and MKNE Group at baseline, 6-weeks, and 3-months post-intervention.

**Figure 3:**
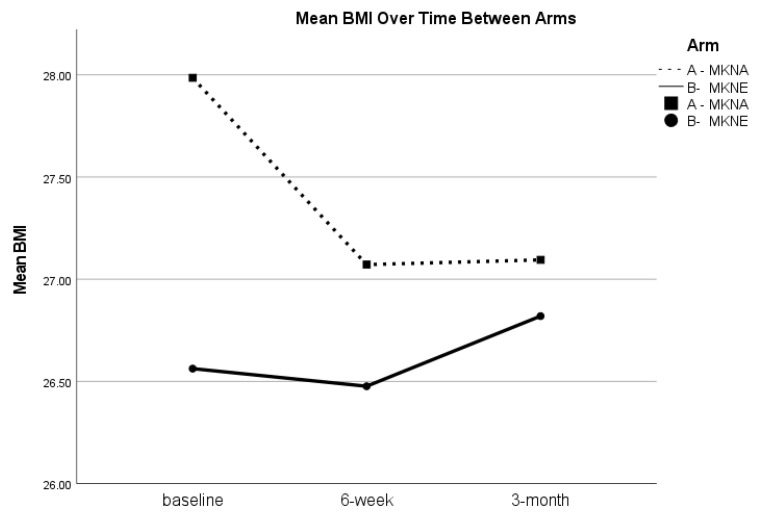
Changes in BMI. RBANS = Repeatable Battery for the Assessment of Neuropsychological Status Indexes; Changes in mean RBANS total scale score for the MKNA and MKNE Group at baseline, 6-weeks, and 3-months post-intervention.

Although blood samples were not collected at the 3-month assessment, cognitive outcomes, BMI, and blood pressure were examined for the full sample from baseline to 3-months, as well as between group differences in change. There were no differences in RBANS total scores or Delayed Memory index scores from baseline to 3-months (*p* > 0.05), suggesting regression to baseline levels during the 3-month post-intervention period. See Figure 2 for RBANS total score trends. Similarly, paired samples t-tests showed that improvements in blood pressure (*p* = 0.072) and BMI (*t*(48) = 1.06, *p* = 0.293) were not statistically significant; however, as seen in Table 3, BMI at 3-months remained lower than baseline levels in the overall sample. Further, while there were no statistically significant between group differences in change from baseline to 3-months for blood pressure or BMI, there was a notable trend for BMI to remain lower in the MKNA group (mean change of −0.82), compared to the MKNE group (mean change of 0.26; *t*(47) = −1.87, *p* = 0.068). See Figure 3.

## 4. Discussion

Reviews of the literature demonstrate that Mediterranean and ketogenic diets provide benefits for cognitive functioning in older adults and may reduce risks for ADRD [4,7,8]. Scalable behavioral interventions are needed to address existing barriers to adherence to MKN, such as education, motivation, and costs. This pilot clinical trial built on prior work to evaluate the feasibility and acceptability of an MKNA program designed to help participants overcome these challenges. Further, we evaluated the influence of the MI-BCT components on adherence and clinical outcomes, as well as overall clinical responses to MKN. Overall, we found that our protocol was highly acceptable and feasible with minor changes. The MI-BCT components were helpful for enhancing adherence and retention in the program, and MKN evidenced clinical benefit for participants while engaged in the program. Implications of these findings and limitations are discussed below.

### 4.1. Feasibility and Acceptability

The pilot demonstrated high feasibility and acceptability of the MKNA program, although a significant area of weakness was identified in our recruitment of a racially diverse sample. Participants in the MKNA arm met our feasibility success criteria, achieving greater than 80% retention in the trial and 90% session attendance, while the MKNE group fell below these thresholds. These differences demonstrate the importance of incorporating MI-BCT strategies in the program to help retain participants in the trial and to increase adherence to the intervention. Across both arms, however, a majority of participants indicated that the quality of the programs was “excellent,” with no significant differences between arms in quality ratings. We believe this finding provides evidence that both arms received a high quality, MKN program.

### 4.2. Adherence

Across the 6-week program, participants in the MKNA arm reported significantly higher self-reported adherence and tended to demonstrate higher levels of urine ketones compared to the MKNE arm. Both arms of the trial demonstrated a substantial drop in self-reported adherence and objective measures of ketones by the 3-month post-assessment. These findings demonstrate that the MI-BCT strategies appear effective for enhancing adherence during the active program but may not confer lasting benefits for remaining in ketosis. These findings are consistent with reviews showing that MI and BCT strategies are effective for increasing adherence to lifestyle programs [20]. However, as noted in a recent systematic review, studies examining integrated MI and behavior change techniques for lifestyle often lack an extended follow-up period to show improvements in long-term adherence [20]. Importantly, other nutrition programs have shown that more resource-intensive (e.g., requiring a dietician and individualized meal plans) and extended programs (e.g., 6-months) can produce sustained adherence to a Mediterranean diet [21]. Thus, to retain scalability, while enhancing long-term adherence, it may be important to extend the number of sessions or incorporate booster sessions beyond the current 6-week MKNA program. Incorporation of additional social support structures, such as an ongoing community support groups for nutrition or online forums for participants to connect, may provide a feasible and scalable way to extend the current program.

In addition to long-term adherence strategies, characteristics of the sample related to adherence may be important to consider for future tailoring. Most characteristics examined were not associated with adherence measures, although a significant limitation for interpreting these results relates to poor racial and ethnic diversity in the sample. Despite this limitation, we found that younger participants with higher cognitive scores at screening tended to report better adherence and evidenced higher levels of ketones during the program. These findings may suggest that earlier intervention targeting younger and cognitively normal individuals with other risks for ADRD may lead to greater benefits from the MKNA program. The strategy of identifying participants in mid- and late-life based on demographic and cardiovascular risks associated with ADRD has been successful in other lifestyle intervention trials [22,23,24,25]. These studies use the Cardiovascular Risk Factors, Ageing and Dementia (CAIDE) risk score as a method for identifying high risk [22,24,25] but otherwise cognitively normal participants into lifestyle intervention trials. To further refine the program, future trials may evaluate effectiveness based on age and cognitive functioning in addition to incorporating qualitative participant feedback from a racially and ethnically representative sample.

### 4.3. Clinical Outcomes

Overall, the MKN programs appeared to benefit participant global cognitive performance, as well as performance on delayed recall, a strong neuropsychological indicator of Alzheimer’s disease risk [26]. Further, across the program participants evidenced improvements in systolic blood pressure and BMI, although blood glucose, HbA1c, and total cholesterol levels were not affected. These findings are consistent with previous studies demonstrating the benefits of Mediterranean and ketogenic diets [9,27,28,29]. In regard to lack of change in blood and serum biomarkers, it is important to note that participants tended to evidence values in or close to the normal range at baseline, while baseline mean BMI and systolic blood pressure scores were above the recommended ranges. Thus, the lack of change in already normal biomarkers and improvements in BMI and systolic blood pressure may be expected and provide additional evidence for the safety and efficacy of MKN for relatively healthy older adults. Similarly, while participants evidenced improvements in cognitive scores, participants were generally performing in the normal range at baseline, which may limit sensitivity to measuring cognitive benefits from the program. By three months post-intervention, a majority of the clinical benefits had regressed to baseline levels. A maintained small decrease in BMI was notable, however, as this demonstrates that participants generally did not experience rebound weight gain on average following the program. In sum, these clinical findings demonstrate that the MKN programs provide clinical benefit while participants are adherent, but these benefits appear to diminish as adherence declines following the active intervention. These findings further support the need for identifying scalable methods for extending aspects of the program to improve long-term adherence. 

### 4.4. Limitations and Future Directions

There are some important limitations to acknowledge when interpreting the findings from this pilot. First, given the relatively small sample size, it is necessary to emphasize that participant attrition may limit generalizability and interpretation of the clinical outcomes reported. Our study was not powered to evaluate moderation effects, which may be an important next step for better understanding for whom and under what conditions MKN is most beneficial. Importantly, however, our results are consistent with the indicators of success based on the NIH ORBIT model for Phase II development and provide a green light for moving forward with intervention development in Phase III.

Second, our inclusion criteria primarily focused on memory complaints and objective scores on a brief cognitive screening tool administered via telephone. As previously noted, this screening protocol may have led to recruitment of a sample with few cardiometabolic risks and relatively normal cognitive functioning, which may have limited our ability to detect clinically important changes on these factors. Protocols recruiting based on multiple risk factors for ADRDs (e.g., demographics, memory, and cardiometabolic) may provide a stronger participant pool for understanding risks and benefits of MKN for high-risk individuals. Further, it may be necessary to increase the number of long-term data collection assessments in order to better evaluate long-term effects of the program and MKN.

Poor racial and ethnic representation in the current sample is another significant limitation that potentially limits the generalizability of our findings. An important future step in the refinement of the MKNA program will involve the use of community engaged research methods and recruitment strategies to increase the diversity of our sample. This issue is especially important in the context of ADRD and related risks, as there is strong evidence that black and Latino individuals [30,31], as well as rural and economically disadvantaged individuals, are at the greatest risk for ADRD and related health conditions [32,33]. In line with recruiting a diverse cohort, it will be important to engage these participants to better understand how the program can be tailored based on location or cultural preferences.

Finally, in order to address scalability of the MKNA program, an important next step in intervention development will involve increasing the structure and simplicity of presentation materials. Currently, the program is led by a clinical psychologist and nurse practitioner, which may not be feasible in many low-resource settings. For example, recorded videos of the nutrition education content, incorporating stop-points, and additional slides for leading guided discussions could provide a straight-forward format without need for extensive specialized training. This modification may be necessary to ensure that the program can be sustainably administered with high fidelity in a community setting by a community health worker.

## 5. Conclusions

This pilot clinical trial provided evidence of the feasibility and high acceptability of the MKNA program. Further, MKNA was associated with short-term benefits for cognition, BMI, and systolic blood pressure. The MI-BCT components were associated with improved retention and increased participant engagement in the trial. Further, MI-BCT strategies appeared to promote greater adherence to MKN. The pilot also elucidated aspects of the program and protocol that will be addressed in future studies, including a need to extend the program beyond six sessions, using new recruitment strategies to increase diversity, and identifying higher risk participants.

## Figures and Tables

**Table 1:** Baseline Demographics.

Baseline Characteristic	*N*	%	*M*	*SD*
Age (years)	58	-	72.91	6.35
Sex				
Female	47	81.0	-	-
Male	11	19.0	-	-
Race				
White	46	79.0	-	-
Black	9	16.0	-	-
Other	3	5.0	-	-
Highest Grade Completed				
High school	3	5.3	-	-
Some college (no degree)	12	21.1	-	-
A.S (or trade school)	4	7.0	-	-
Bachelor’s Degree	14	24.6	-	-
MS (or equivalent postgrad)	18	31.6	-	-
PhD, MD (or equivalent)	6	10.5	-	-

Note. *N* = 58. *M* = mean; *SD* = standard deviation.

**Table 2:** Means and Standard Deviations of Primary Adherence Outcomes Between Arms.

Outcome	*N*	MKNA Group	*N*	MKNE Group	*p*-Value
Adherence rating across 6-weeks	25	5.97 (2.53)	24	4.49 (2.21)	0.034 *
6-week ketones ^a^	25	17.32 (14.41)	24	10.54 (12.83)	0.089
Self-reported adherence (3-month)	26	3.50 (2.99)	23	3.35 (2.77)	0.854
Ketone levels (3-month)	23	1.09 (2.11)	22	1.14 (3.43)	0.681

Note. *N* = 58. Standard deviations are in parentheses. ^a^ ketones = Mean days above trace levels across the 6-week intervention. * *p*< 0.05, two-tailed.

**Table 3:** Mean Changes in Clinical Outcomes Over Time Across Arms.

	Baseline *M* (*SD*)	6-Week *M* (*SD*)	3-Month *M* (*SD*)	% Change ^a^ (Baseline to 6-Weeks)	% Change ^b^ (Baseline to 3-Month)
RBANS total scale score	102.46 (14.75)	105.00 (15.15)	101.55 (14.95)	+2.48%	+1.03%
Delayed Memory Index score	101.71 (17.22)	106.15 (13.80)	103.05 (12.02)	+4.37%	+2.21%
Ketones (mg/dL)	1.33 (2.24)	8.49 (15.12)	1.25 (2.94)	+538.35%	±0.00%
HbA1c	5.21 (0.42)	5.29 (0.52)	-	+1.55%	-
Blood glucose	110.17 (19.43)	110.15 (19.01)	-	−0.018%	-
Total cholesterol	198.00 (55.00)	211.46 (68.69)	-	+6.80%	-
Systolic blood pressure	136.66 (20.72)	130.22 (20.81)	130.52 (19.17)	−4.71%	−4.04%
BMI	27.62 (5.67)	27.02 (5.42)	26.81 (5.41)	−2.17%	−2.93%

Note. *N* = 58 at baseline; *N* = at 6-weeks; *N* = at 3-months. RBANS = Repeatable Battery for the Assessment of Neuropsychological Status; HbA1c = Hemoglobin A1c; BMI = body mass index. ^a^ 6-week minus baseline; ^b^ 3-month minus baseline.

## Data Availability

The data presented in this study are available on request from the corresponding author. The data are not currently publicly available due to privacy restrictions.
